# Optimization of the Bacterial Cytochrome P450 BM3 System for the Production of Human Drug Metabolites

**DOI:** 10.3390/ijms131215901

**Published:** 2012-11-28

**Authors:** Giovanna Di Nardo, Gianfranco Gilardi

**Affiliations:** Department of Life Sciences and Systems Biology, University of Torino, via Accademia Albertina 13, 10123 Torino, Italy; E-Mail: giovanna.dinardo@unito.it

**Keywords:** cytochromes P450, drug metabolite, active metabolite, toxicity test, adverse drug reactions

## Abstract

Drug metabolism in human liver is a process involving many different enzymes. Among them, a number of cytochromes P450 isoforms catalyze the oxidation of most of the drugs commercially available. Each P450 isoform acts on more than one drug, and one drug may be oxidized by more than one enzyme. As a result, multiple products may be obtained from the same drug, and as the metabolites can be biologically active and may cause adverse drug reactions (ADRs), the metabolic profile of a new drug has to be known before this can be commercialized. Therefore, the metabolites of a certain drug must be identified, synthesized and tested for toxicity. Their synthesis must be in sufficient quantities to be used for metabolic tests. This review focuses on the progresses done in the field of the optimization of a bacterial self-sufficient and efficient cytochrome P450, P450 BM3 from *Bacillus megaterium*, used for the production of metabolites of human enzymes. The progress made in the improvement of its catalytic performance towards drugs, the substitution of the costly NADPH cofactor and its immobilization and scale-up of the process for industrial application are reported.

## 1. Introduction

Drug discovery is a long and expensive process requiring many different steps starting from formulation up to preclinical and clinical trials [ 1]. Two of the main causes of human drug failures are lack of effectiveness and safety [ 2]. For this reason, it is important to understand at early stages how fast a drug is metabolized to assess its effectiveness, as well as to identify and test all the metabolites for their possible biological activity.

In certain cases, a metabolite can have the same effect of the parent drug. For example, one of the metabolite of propranolol, a non-selective β-blocker used to treat hypertension, anxiety and panic, is 4′-hydroxypropranolol. This compound has been demonstrated to have similar effects of propranolol as a β-receptor antagonist [ 3] and it is believed to contribute to the bioactivity of this drug [ 4]. Other monohydroxylated products of this drug have also been reported to be active as β-blockers [ 5].

Since the early 70s, it has been known that active metabolites can cause the so-called adverse drug reactions (ADRs) [ 6–8]. For example, it has been widely demonstrated that bioactivation of drugs can cause DNA modification and relevant mutations for cancer developmet [ 6]. The toxic effects of drugs became known in 1973, concerning the anti-inflammatory agent and hepatotoxic acetaminophen [ 9]. It was then demonstrated that a reactive quinone–imine intermediate derived from a cytochrome P450 mediated oxidation of this drug was able to deplete the endogenous antioxidant glutathione (GSH) and/or to bind covalently liver proteins leading to hepatic toxicity [ 10].

It also has to be taken into account that some drugs are administrated as pro-drugs and they are not biologically active if not converted into an active metabolite. This is the case of terfenadine that is converted into fexofenadine, a molecule active as non-sedating histamine H1 receptor antagonist [ 11].

All these considerations point out that safety issues are crucial for drug approval by the FDA, and that not only the drug candidate has to be tested for toxicity, but also the effects of its metabolites must be known to avoid ADRs. Such studies can require large quantities of the pure metabolites, and these may be difficult to synthesize by conventional chemical methods.

## 2. Phase I Drug Metabolism

Cytochromes P450 (CYPs) are a large superfamily of heme-containing monoxygenases mainly catalyzing C–H hydroxylations on endogenous and exogenous compounds [ 12]. Though hydroxylation is the predominant catalyzed reaction, many other different oxidations can be performed by these enzymes on a wide range of structurally different substrates [ 13–15]. This makes it difficult to predict *a priori* the reaction product generated from a given substrate.

Human cytochromes P450 are enzymes anchored to the endoplasmic reticulum (ER) via a *N*-terminal sequence [ 16, 17] and they require a redox partner, cytochrome P450-reductase (CPR) to perform their function [ 18]. CPR is also anchored to the ER membrane, and its role is to transfer the electrons from NADPH to the heme cofactor of the P450. In human liver, cytochromes P450 play a predominant role in phase-I drug metabolism and clearance since they turn over the large majority of the known commercially available drugs into one or more metabolites [ 13].

The isoforms CYP3A, CYP2D6, CYP2C, CYP1A2 and CYP2E1 are responsible for the metabolism of 80% of the clinically used drugs [ 19, 20]. However, different metabolites can be produced by one or more isoforms of cytochromes P450 and the metabolites produced by one enzyme can be the substrates for a different isoform of P450. As a consequence the metabolite profile of a drug can be quite complex taking also into account that different oxidative reactions can take place on different attack positions, depending also on the orientation of the drug in the active site of the P450 enzyme.

Two key steps are required during drug discovery: the first is the identification of all the metabolites produced for a given drug [ 21], and the second is the synthesis of these compounds for toxicity tests.

The need to deal with complex metabolite profiling in conjunction with a number of human cytochromes P450 has led to the creation of a microfluidic integrated electrochemical system that presents the added advantage of the use of 30 μL of drug solution, with the attractive possibility of further miniaturization. The use of an autosampler as the pumping system performs the double role of increasing the repeatability and controlling the flow with the added potential of ease of integration in the robotic high throughput system currently available in the pharmaceutical industry. The system allows us not only to electrochemically determine kinetic parameters such as the *K*_M_ of a drug for a given P450, but it also makes possible the product identification by HPLC-MS [ 22].

Chemical methods are often difficult to develop for certain metabolites and are not cost-effective. An alternative to chemical synthesis is to use cytochromes P450 to generate the metabolites of drugs. For this reason, human liver microsomes or human P450 enzymes heterologously expressed in bacteria [ 23, 24] and in insect cells [ 25], and purified have been employed as potential candidates for biocatalysis to prepare sufficient quantities of human drug metabolites for toxicity tests. In general, the engineering of mammalian P450s as versatile biocatalysts for many biotechnological applications has been reported [ 26–31]. However, hepatic microsomes have limited availability and highly variable expression levels of cytochromes P450 whereas the human purified enzymes show low catalytic activity and poor stability for industrial use [ 32, 33]. One of the solutions to this issue is the use of other P450 enzymes as surrogate for the synthesis of human metabolites. This will be the focus of this review.

## 3. P450 BM3 as Biocatalyst

**Figure 1:**
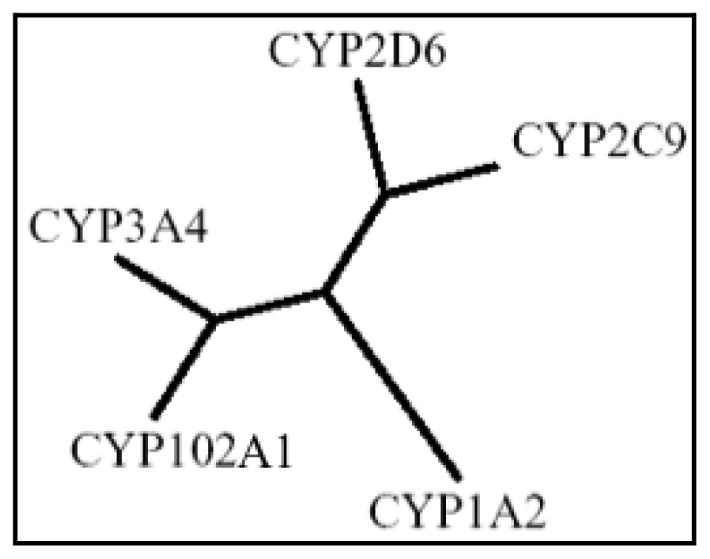
Phylogenetic tree based on the sequences of P450 BM3 (CYP102A1) and the major human isoforms involved in drug metabolism. The unrooted phyolgenetic tree was built aligning the substrate recognition sites (SRS) of the chosen P450s.

Cytochrome P450 BM3 from *Bacillus megaterium* can offer a solid alternative for the synthesis of drug metabolites at industrial scale. In fact, the enzyme (CYP102A1) is a soluble and self-sufficient enzyme with a diflavin-containing reductase fused to a heme-containing P450 domain in a single polypeptide chain. It has been discovered in *Bacillus megaterium*[ 34] and it acts as a fatty acids oxygenase displaying a high activity (>1000 turnovers/min) and nearly 100% coupling efficiency [ 35, 36]. It can be expressed at high levels in recombinant *E. coli* and it is able to turn over different substrates. In 2007, our group demonstrated the ability of wild-type P450 BM3 to turn over different drugs [ 37]. We used a screening assay procedure developed in our lab, the so-called alkali assay [ 38], to test the ability of the purified enzyme to turn over different drugs usually metabolized by different subfamilies of human P450s. Some of the drugs resulted positive to the assay and were selected for further studies aimed to identify the metabolites. The enzyme is able to perform different reactions on different drugs, including N-dealkylation of propranolol, hydroxylation of chlorzoxazone and dehydrogenation of nifedipine [ 37]. Phylogenetic analysis shows that P450 BM3 (CYP102) is very close to the human counterparts, specifically P450 3A4, when considering the main human enzymes involved in drug metabolism (Figure 1).

Very recently, we also tested a small library of 1,2,5-oxadiazole derivatives, a class of drug candidates acting as NO pro-drugs donors for the treatment of cardiovascular diseases on the wild-type protein and nine of them resulted to be turned over by the bacterial enzyme, with coupling efficiency ranging from 55% to 100% [ 39].

The high efficiency and versatility of P450 BM3 are the main reasons why it is considered a prototype for a biocatalyst with human P450 activities [ 40–42] offering a solid scaffold for biotechnological applications [ 43] including drug metabolite production [ 44].

## 4. Optimization of P450 BM3 as a Biocatalyst for Drug Metabolites Production

**Figure 2:**
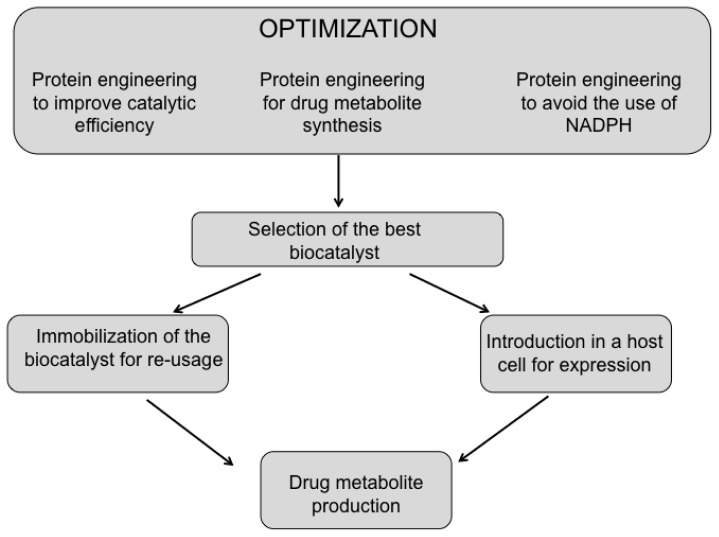
Steps for the application of P450 BM3 as biocatalyst for the synthesis of drug metabolites.

The exploitation of the bacterial enzyme as biocatalyst for the production of drug metabolites requires a multi-step approach aimed at the optimization of the enzyme performance by protein engineering and the development of a platform allowing the regeneration/avoidance of the NADPH cofactor and the immobilization for re-usage of the biocatalyst to reduce costs (Figure 2).

Thus far the scientific literature has been focused on the following goals:

1. The increase of the substrate specificity by protein engineering;
2. The improvement of the catalytic performance (K_M_, k_cat_, coupling efficiency) of P450 BM3 toward drugs;
3. The substitution of the costly NADPH cofactor;
4. The immobilization and scale-up of the process for industrial application.

The achievements in these fields of endeavor are reported here.

### 4.1. Protein Engineering to Improve Substrate Selectivity

The availability of the crystal structure of the heme domain of P450 BM3 in the substrate-free form [ 45] and in complex with the palmitoleic acid [ 46] offers the opportunity to identify key residues for substrate binding and catalysis and therefore to carry out mutagenesis experiments. Molecular docking simulations are also a very useful tool to predict if a drug would enter the catalytic pocket of the bacterial enzyme and to understand how the substrate should be oriented in the active site for hydroxylation.

Mutagenesis has been extensively used on P450 BM3 to introduce new abilities to metabolize drugs or to improve the existing ones. The strategies used are site-directed mutagenesis, site-saturation mutagenesis, directed evolution or a combination of the three approaches. Different metabolites of different drugs have been produced and identified using a panel of variants, some of them sharing some critical mutations. The variants generated up to now cover a wide range of structurally different drugs and often give rise to different metabolites, summarized in Table 1, with different yield. The availability of several crystal structures of P450 BM3 mutants [ 47] gives also the opportunity to perform docking simulations and to have important information not only on how to further improve the biocatalyst but also, more generally, on the structure-function relationship of the enzyme.

A first experimental evidence that cytochrome P450 BM3 can bind drug-like molecules came in 2005 from the work of Van-Lussenburg and co-workers [ 48]. A panel of variants of the bacterial enzyme was generated and showed the ability to turn over the fluorescent substrates alkoxyresorufins. The alkoxyresorufins *O*-dealkylation assay allowed to test 45 drug-like molecules and demonstrated for the first time that variants of the bacterial enzyme can be inhibited by drug-like molecules and therefore can bind them [ 48].

In 2006, site saturation mutagenesis toward selected active site residues (A74, L75, V78, P81, A82, F87, and T88) was applied in combination with random mutagenesis and allowed the generation of a mutant able to produce three metabolites of the drug propranolol (4-hydroxy-, 5-hydroxy- and *N*-despropylpropranolol) [ 78]. If we consider that the wild type enzyme is able to produce only one metabolite corresponding to the *N*-desproylated form of propranolol [ 37], this is a very good demonstration of how the bacterial P450 BM3 is an optimal template for protein engineering and a versatile enzyme able to perform different reactions and attack different positions.

Site-directed mutagenesis was also used to create a panel of mutants of P450 BM3, able to metabolize probe substrates for human cytochromes P450 such as 7-ethoxycoumarin that is widely used to measure the drug metabolizing activity in liver and testosterone, that is widely used to probe the activity of P450 3A4. The P450 BM3 variants were shown to perform 3-hydroxylation and *O*-deethylation, producing the typical metabolites of human enzymes [ 41]. The rates for the two reactions were increased by up to 61- and 129-fold with respect to wild type for the *O*-deethylation and 3-hydroxylation reactions, respectively.

In 2006, the triple mutant R47L/F87V/L188Q was found to metabolize testosterone as well as other molecules such as amodiaquine, dextromethorphan, acetaminophen, and 3,4-methylenedioxy-methylamphetamine (MDMA) [ 49]. Testosterone was converted into three metabolites that were found to be monohydroxylated derivatives of the drug. One of them resulted to be 16β-hydroxytestosterone whereas 6β-hydroxytestosterone, the major metabolite formed by human P450 3A4 [ 73], was not detected. However, very recently, a single mutation (A82W) introduced in two previously generated P450 BM3 variants was reported to improve the regioselectivity toward steroid hydroxylation in position 16β [ 84]. Furthermore, the single active site mutation S72I introduced in the same variants resulted to invert the stereoselectivity producing 16α-hydroxytestosterone [ 85]. These data are again an excellent example of how the bacterial enzyme can be manipulated toward the desired and enantiospecific function.

The triple mutant R47L/F87V/L188Q showed also new catalytic abilities with respect to the wild-type enzyme concerning the conversion of amodiaquine into two metabolites identified by MS analysis as *N*-desethylamodiaquine and a monohydroxylated metabolite [ 49]. Also dextromethorphan resulted to be *N*-demethylated to form the metabolite 3-methoxymorphinan and MDMA was metabolized by the bacterial triple mutant to three metabolites: 3,4-methylenedioxyamphetamine (MDA), *N*-hydroxy-3,4-methylenedioxym-ethylamphetamine (N-OH-MDMA) and 3,4-dihydroxymethylamphetamine (3,4-OH-MA). Acetaminophen was also metabolized by P450 BM3 R47L/F87V/L188Q to the *N*-acetyl-*p*-benzoquinoneimine (glutathione conjugate) [ 49]. The rates of product formation resulted lower than those measured for the human enzymes for the same metabolites, but the triple mutant was then used as a template for a round of random mutagenesis to create a new library of variants. Two more rounds of random mutagensis allowed the generation of four mutants (M01, M02, M05 and M11) with improved activity toward dextromethorphan and MDMA in comparison to the starting variant [ 53]. The product formation was increased up to 200-fold for dextromethorphan when compared to the triple mutant and the activities of the random mutants resulted up to 90-fold higher than the values reported for human P450 2D6 [ 53]. In this work, three key mutations were identified (F81I, E267V, L86I) and, interestingly, one of them (L86I) was not located in the active site.

The four mutants M01, M02, M05 and M11 were also found to generate the same GSH adducts of diclofenac, as the one obtained with incubations with human liver microsomes [ 62, 65]. In particular, P450 BM3 M11 yielded similar oxidative metabolite profiles of diclofenac as human P450s, producing also 4′-hydroxy- and 5-hydroxydiclofenac. This mutant was therefore expressed in yeast that was used as model system for toxicity studies on the metabolites of diclofenac, a drug known to induce severe liver toxicity [ 86]. The results showed that yeast strains expressing P450 BM3 M11 grew significantly slower in the presence of diclofenac than the control strains not expressing the P450 enzyme. The metabolites 4′- and 5-hydroxydiclofenac did not show any effect on cell growth in cells expressing P450 BM3 M11, suggesting that they are not involved in yeast toxicity [ 49]. This work represents a pioneer study in a model system; however it demonstrates the feasibility to use bacterial P450 BM3 variants also directly in cells to perform toxicity tests.

**Figure 3:**
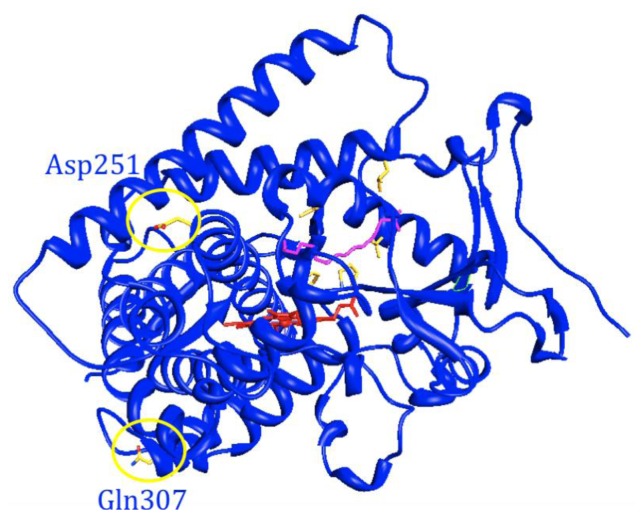
Structure of the heme domain of P450 BM3 in complex with the substrate palmitoleic acid (magenta) [ 46]. The yellow circles indicates the residue mutated in the variant A2, that can metabolize diclofenac, ibuprofen and tolbutamide. The heme is shown in red.

In a recent work of our group, a mutant carrying two mutations generated by random mutagenesis, showed the new abilities to metabolize diclofenac, ibuprofen and tolbutamide to 4′-hydroxydiclofenac, 2-hydroxyibuprofen and 4-hydroxytolbutamide, respectively [ 66]. The mutant, named A2, also in this case carries the two mutations far away from the active site (Figure 3) and it is very interesting since it can generate the human metabolites of three drugs usually metabolized by CYP2C subfamily in human liver.

Other variants able to convert ibuprofen were generated by directed evolution [ 47]. These variants showed also a new activity toward desmethylnaproxen that is the human metabolite of naproxen, another substrate of CYP2C9 *in vivo*[ 47]. It is remarkable that all these data suggest indeed that it is possible to produce different bacterial mimics of each human P450 both in terms of substrate specificity and metabolic profile. Furthermore, the crystal structure of an early intermediate in directed evolution experiments was solved and showed the effect of a critical residue of the active site (L75R) in increasing the flexibility of B′-helix [ 47]. It is therefore important to use also random approaches to engineer the bacterial enzyme to introduce mutations that change the flexibility of the protein that can be crucial to accommodate new substrates. Often changes in flexibility are due to mutations far away from the active site and therefore unpredictable by rational design approaches. The other advantage of directed evolution approaches is the generation of libraries of mutants, increasing the chance to obtain variants that perform multiple reactions including the desired one. This was the case of the generation of 43 mutants active toward verapamil [ 55], a calcium channel blocker used in the treatment of hypertension and arrhythmia [ 87]. The variants derived from mutants previously generated for the conversion of propranolol [ 78] and for stereoselective and enantioselective alkane hydroxylation [ 88, 89]. Some of the variants were chimeric forms derived from the recombination of CYP102A1 with other bacterial enzymes belonging to the CYP102A subfamily (see paragraph 4.2). Ten different metabolites were produced and six of them are the ones typically produced by human P450 3A4 [ 90]. In the work of Sawayama and co-workers [ 55], 42 variants active toward astemizole, a potent H1-histamine receptor antagonist used for treatment of common sinus allergy symptoms, were also generated producing seven different metabolites, four of them produced by human enzymes [ 80].

In the same work, variants able to metabolize a third compound, LY294002, an antiproliferative agent that inhibits phosphatidylinositol 3-kinase [ 91] were also generated and produced 2 hydroxylated products also produced by human enzymes. Interestingly, the LY294002 conversion by the variants resulted lower (<15%) than the ones obtained for verapamil (85%) and astemizole (85%). However this trend correlates with the profile obtained with rat liver microsomes, demonstrating that the compound LY294002 is less prone to C–H oxidation catalyzed by P450 enzymes than verapamil and astemizole [ 91]. These data point out one of the other advantages in the use of bacterial P450s as surrogate of the human enzymes, sharing general reactivity features.

It has to be taken into account that enantioselectivity is often a very important requirement for the synthesis of some drug metabolites and it often represents a limit of chemical synthetic methods. However, this kind of selectivity has been achieved by the work mentioned above [ 55] where a single mutation resulted to invert the enantioselectivity for 16-testosterone. Furthermore, a mutant of P450 BM3 able to metabolize buspirone into the metabolite (*R*)-6-hydroxybuspirone that was the sole product [ 57] was also produced and a high yield of conversion achieved (72% in 7 h).

Human chiral metabolites of simvastatin and lovastatin were also obtained by wild type P450 BM3 and the activity toward these two drugs was increased in two variant carrying six and seven mutations, generated by site-directed mutagenesis [ 70]. These two drugs are used to treat hyperlipidemia and hypercholesterolemia and are usually oxidized by human CYP3A4/5 to several products, including 6′b-hydroxy, 3-hydroxy- and exomethylene metabolites. The mutants not only resulted to produce the correct chiral compound but also showed a catalytic efficiency higher than the one reported for human P450 3A4 with a *k*_cat_/*K*_m_ up to 7-fold higher than the human enzyme [ 70].

P450 BM3 variants were found to metabolize clozapine, an antipsychotic drug and acetaminophen, a widely used analgesic and antipyretic drug, at significantly higher (up to 70-fold) levels than human and rat microsomes [ 62]. This drug is mainly metabolized by human P450 1A2 [ 92]. The oxidation of other substrates metabolized by P450 1A2 was achieved also by site directed mutagenesis. Mutants with improved activities toward phenacetin, ethoxyresorufin and methoxyresorufin respect to the wild type enzyme were generated and the *O*-deethylation product of phenacetin obtained [ 75].

The increasing need to identify new advantageous mutations for the production of metabolites from drugs covering a large fraction of the chemical space led to the design of an experimental strategy aimed at the generation of a minimal set of BM3 mutants with differences in regio- and stereoselectivities toward a library of drugs [ 51]. In this work, drug depletion by five different mutants was observed for 43 molecules. Furthermore, LC-MS analysis performed in selected single ion modes allowed the identification of multiple metabolites for nine drugs. Some of them are produced also by human liver microsomes (HLM) whereas others resulted specific for P450 BM3 variants, able to produce up to 13 different metabolites for ondansetron. Interestingly, the metabolite profile for amitriptyline, buspirone, ondansetron, propafenone, and repaglinide was different for the five variants studied, allowing the identification of key mutations changing the regio-specificity [ 51].

### 4.2. Protein Engineering to Improve Catalytic Efficiency

Although the protein engineering work of the last 7 years has been successful in producing several mutants of P450 BM3 able to turn over different drugs into different metabolites, much work is still needed to improve the catalytic performance of the potential biocatalysts. One of the most obvious tools will be to combine the different mutations now available and to introduce, where not present, the crucial changes in those positions known to improve the catalytic efficiency of the enzyme such as R47, Y51, F87 and A328V [ 93, 94].

Furthermore, different approaches of directed evolution such as DNA shuffling, already successful in the evolution of P450 enzyme (see for example [ 95– 99]) could give rise to large libraries of mutants with an increased possibility to select the desired functions. Such approaches can include different bacterial homologous P450 fusion proteins such as CYP102A5 and A7 that have already been engineered to oxidise chlorzoxazone and diclofenac [ 100]. A chimeric library of synthetic P450s has already been created from the recombination of different homologous bacterial enzymes, CYP102A1, CYP102A2 and CYP102A3 [ 57, 78, 101]. The authors used a powerful structure-based algorithm called SCHEMA to identify fragments of proteins that can be recombined to minimize disruptive interactions that would prevent protein folding [ 101]. The heme domains generated by recombination were also fused with the three different reductase available. This approach has been used to generate a synthetic family of 16 P450 BM3 heme domain variants fused to three different reductases [ 57]. A cluster of chimeric enzymes showed high activity toward tolbutamide, propranolol and chlorzoxazone.

Since the catalytic performance of P450 BM3 is also highly dependent on the electron transfer efficiency from NADPH to the substrate via the fused reductase containing the two flavin cofactors, new chimeric systems can be engineered by fusing at genetic level different domains, an approach known as “Molecular Lego” [ 102]. The so-called “optimized chimeragenesis” [ 103], a combination of synthetic P450s fused to different redox partner, can therefore offer a powerful tool to improve and optimize the biocatalytic properties for drug metabolite production.

A very interesting observation was also made by the group of van Vugt-Lussenburg and co-workers [ 49]. They noticed that the metabolism of 3,4-methylenedioxymethylamphetamine and acetaminophen by P450 BM3 variants could be stimulated up to 70-fold by the addition of caffeine, a known activator of rat P450 3A2. They also observed homotropic cooperativity with testosterone and our group observed the same behaviour with the mutant A2 toward ibuprofen [ 66]. These data suggest that heterotropic and homotropic cooperativity, previously described for human P450 3A4 [ 104–110] can also take place in P450 BM3 [ 49]. One of the possible explanations for these observations is that the mutants can accommodate more than one molecule in the active site and that the one molecule forces the other into a specific orientation, reduces substrate mobility and therefore triggers the reaction.

The level of coupling for drug metabolite production by P450 BM3 variants has not been widely investigated until now, due to the fact that the attention has been focused to the introduction of new abilities in the bacterial enzyme. Nevertheless, very recently, the level of coupling for testosterone 16β-hydroxylation has been reported to range from 13.5% to 83.5% in different mutants of P450 BM3 [ 84]. Taking into account that the level of coupling for testosterone turnover by human P450 3A4 has been reported to be 16% [ 111], the data mentioned above demonstrate that the bacterial enzyme can retain its coupling efficiency also when metabolizing human substrates.

### 4.3. Substitution of the Costly NADPH Cofactor

The need of the NADPH cofactor to drive electron transfer in the P450 catalysis significantly affects the cost of the scale-up of a biocatalytic process. For this reason, different solutions to this issue have been proposed. A simple and efficient solution uses hydrogen peroxide as oxidant in place of NADPH. In the presence of high peroxide concentrations the catalytic cycle of cytochromes P450 is forced to the peroxide shunt and the P450 works as a peroxide-dependent enzymes such as chloroperoxidase or P450BSb [ 112]. As early as in 1999 mutants of the bacterial P450cam from *Pseudomonas putida* able to hydroxylate naphthalene in the absence of cofactors through the ‘peroxide shunt’ pathway were created by directed evolution [ 113]. The mutants showed an activity improved by 20-fold respect to the wild type enzyme [ 113]. Different studies on P450 BM3 have shown that the enzyme is able to use this pathway to hydroxylate different compounds [ 114, 115]. Later, mutants of P450 BM3 were generated to metabolize propranolol into hydroxylated and *N*-despropylated forms using the peroxode shunt [ 78].

Another opportunity to reduce costs for a continuous use of NADPH is its regeneration by using dehydrogenases. The systems developed for P450 BM3 have been extensively reviewed by Whitehouse *et al.*[ 112]. The authors also reviewed the protein engineering work that has been done on P450 BM3 to accept the less expensive cofactor NADH.

Biomimetic and less expensive cofactors, such as *N*-benzyl-1,4-dihydronicotinamide have also been shown to drive the catalysis of mutants of P450 BM3 [ 116].

An alternative to bypass the use of NADPH is the electrochemical reduction of the P450 enzyme. Despite the full bacterial enzyme or only the heme domain (BMP) have been immobilized on different electrode surfaces [ 117–119], only few reports have shown that the protein is able to turn over substrates by using the reducing equivalents donated by the electrode surface. Our group achieved catalysis on the substrate *p*-nitrophenol with the BMP immobilized on modified glassy carbon electrodes [ 120] and the chimeric protein BMP/Fld, where the redox protein flavodoxin from *Desulfovibrio vulgaris* was fused to BMP, resulted in a 6-fold increase of the amount of the reaction product *p*-nitrocatechol [ 120]. One key factor in electrochemically-driven P450 catalysis firstly reported by our group [ 121, 122], is that strategies for immobilisation of electrode surfaces leading to high electron transfer values do not necessarily lead to catalytically active enzymes. Indeed slow heterogeneus electron transfer values allow for catalytic activity with the formation of product. This point has been reinforced by protein and electrode engineering that demonstrated the coupling efficiency can be modulated by controlling the electron transfer process from the electrode surface to the P450 enzyme [ 123]. These findings demonstrate how important is the control of the electron flow to the heme cofactor for the development of an efficient biocatalyst. Electrochemical catalysis was also achieved by the entrapment of P450 BM3 on polypyrrole on platinum and glassy carbon electrodes, reported to allow catalysis on the substrate p-nitrophenoxycarboxylic acid (pNCA) [ 124].

The need of NADPH was bypassed also by using a mediator such as cobalt(III)sepulchrate and zinc dust that serves as an electron source [ 125].

Other studies where NADPH was regenerated by non-enzymatic methods for monoxygenation reactions have been previously reviewed [ 126].

### 4.4. Immobilization and Scale-Up of the Bioprocess for Industrial Application

The purified engineered P450 can be immobilized on a solid supports allowing the re-usage of the biocatalyst and most probably increasing the stability of the enzyme. In any case, a very stable enzyme is required, able to maintain its activity over time and in a wide range of temperature. For this reason, protein engineering has been aimed to create P450 BM3 variants with increased thermostability [ 127] also in combination with peroxygenase activity [ 128]. In this last case, 44 novel thermostable variants were generated showing half-lives of inactivation at 57 °C up to 108 times that of the most stable parent [ 128]. They were created by the recombination of stabilizing fragments and were demonstrated to metabolize also the drugs verapamil and astemizole [ 128]. Examples of immobilization of P450 BM3 on different supports are present in the literature.

Different matrices were used by Maurer and co-workers to immobilize the entire enzyme. The best method resulted encapsulation in a sol-gel matrix derived from tetraethoxy orthosilicate. The authors used also a cofactor recycling system based on formate dehydrogenase [ 129]. Sol-gel immobilisation of P450 BM-3 improved also enzymatic stability and activity toward b-ionone, octane and naphthalene [ 130].

The heme domain (BMP) of the mutant F87A was immobilized on two different mesoporous molecular sieves and showed activity using hydrogen peroxide as source of electrons and 12-pNCA and n-octane as substrate [ 130]. For this last substrate the activity of the immoblised protein was 2-fold higher compared to the free form.

Recently, a mutant of P450 BM3, was immobilized on a DEAE-650S support and entrapped with k-carrageenan and a mediator, Zn/Co(III)sep, was used instead of NADPH. Zinc dust was used as electron source and catalase to remove hydrogen peroxide [ 131].

In this work, a small-scale bioreactor was developed by immobilizing the mutant of P450 BM3 named M9 carrying 8 point mutations. P450 BM3 M9 was used for the continuous conversion of 3-phenoxytoluene in a plug flow reactor and the reactor was shown stable for 5 days with total turnover numbers over 2000 [ 131].

Attempts have been made to set up whole bacterial cells biotransformations based on P450 BM3. For example, a whole-cell biocatalyst based on cell-surface display of P450 BM3 has been developed. The method uses a fusion system consisting of P450 BM3 and an ice-nucleation protein (Inp) from *Pseudomonas syringae* to display the bacterial enzyme on the surface of *Escherichia coli* cells [ 132]. The surface-displayed P450 BM3 showed a *k*_cat_ value lower than the purified enzyme (65%), but a similar K_m_ value for the substrate 12-pNCA.

*Escherichia coli* BL21 were also use to co-express a quintuple mutant of P450 BM3 and a NADPH regenerating system consisting of a glucose facilitator from *Zymomonas mobilis* for uptake of unphosphorylated glucose and a NADP^+^-dependent glucose dehydrogenase from *Bacillus megaterium*. The biocatalyst was optimized and showed a 9-fold increased product formation rate toward the substrate alpha-pinene oxide [ 133].

The production of drug metabolites by cytochrome P450 BM3 has not yet reached the industrial scale since the limitations described above (cofactor requirement, low turnover rates) have not been totally overcome. Nevertheless, the introduction of the P450 BM3 variants in a host cell and the optimization of the fermentation conditions to obtain high levels of expression of the biocatalyst seems to be the most promising option [ 134, 135]. Furthermore, biotransformations based on bacterial and yeast P450s have already reached the industrial scale [ 33] giving a reference point of yields of production required for a biological process to be exploited at industrial level. It can be noted that pravastatin is produced by hydroxylation of compactin catalysed by CYP105A3 from *Streptomyces* sp. [ 136]. The productivity reported is 15 mg L^−1^ h^−1^.

## 5. Conclusions and Future Perspectives

This review has shown that great progress has been made by protein engineering techniques in enabling the creation of a wide panel of P450 BM3 variants able to recognize and produce many drug metabolites generated by the major human liver P450 isoforms. Though more work is still currently being performed on this enzyme, also other bacterial homologues are becoming the center of studies aiming at increasing the variability of biocatalysts with the desired function. New experimental strategies based on computational methods are making possible the identification of the mutations required for the efficient production of the same metabolites generated by the human liver enzymes. Much work is still needed to increase the performance of these enzymes in terms of turnover and coupling efficiency. These hurdles not only can be overcome, but they also are less important when considering the huge advantages offered by the sustainable green approach offered by the use of engineered enzymes.

## Figures and Tables

**Table 1:** Drugs and corresponding metabolites produced by WT and mutants of cytochrome P450 BM3.

Drug	Structure	Metabolite produced by P450 BM3 WT or variants	Main human P450 involved	Ref.
Acetaminophen	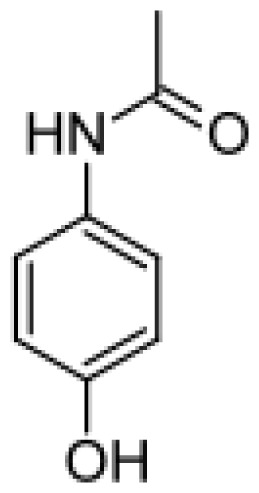	*N*-acetyl-*p*-benzo-quinone imine [49]	2E1	[50]
Amitriptyline	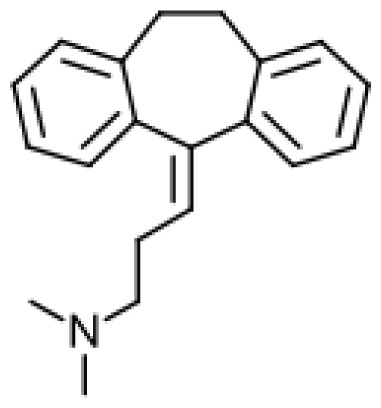	Nortriptyline [51]	2C19, 3A4, 1A2	[52]
Amodiaquine	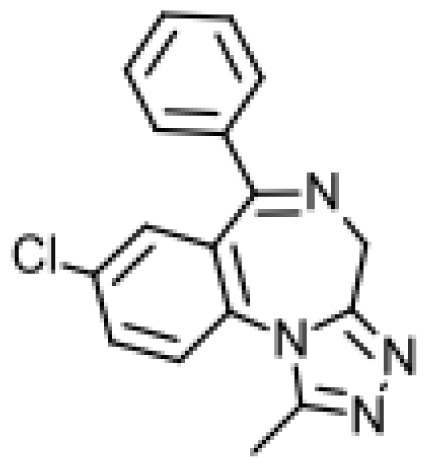	*N*-desethylamodiaquine [53]	2C8	[54]
Astemizole	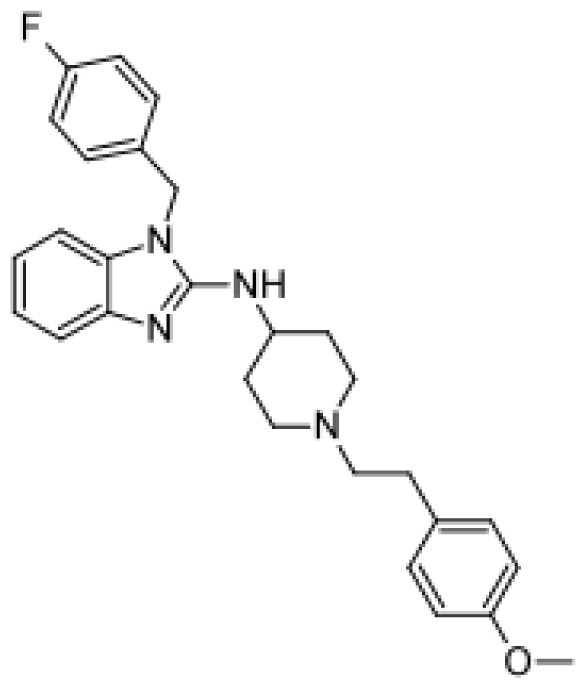	6-hydroxyastemizole [55]	2D6	[56]
		6-hydroxydesmethylastemizole [55]	2D6	
		Desmethylastemizole [55]	2D6	
		Norastemizle [ 55]	3A4	
Buspirone	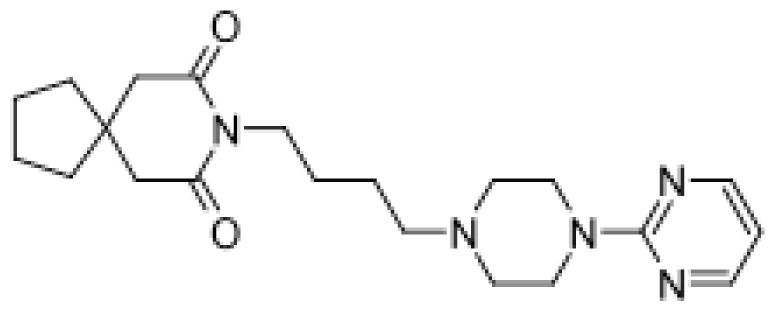	(*R*)-6-hydroxybuspirone [57]	3A4	[58]
		Mono- and dihydroxylated metabolites [51]	3A4	
Chlorzoxazone	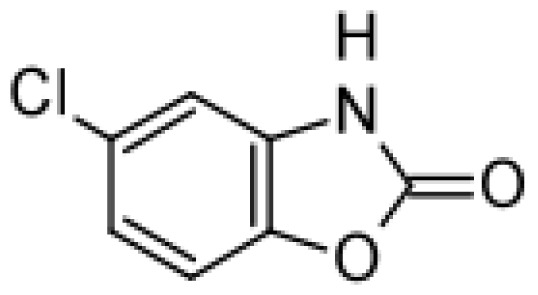	6-hydroxychlorzoxazone [37]	2E1	[59]
Cilostazol	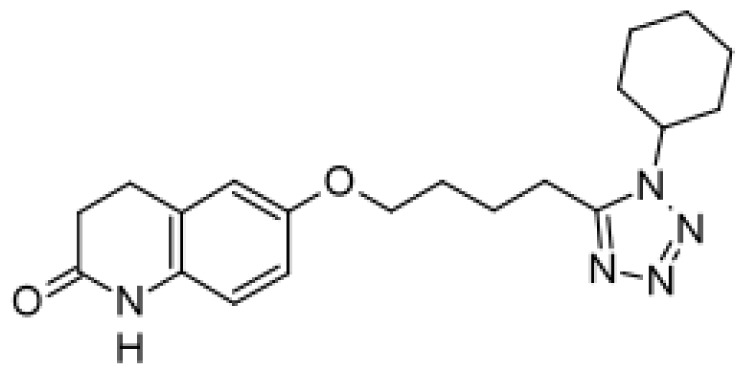	Mono- and di-hydroxymetabolites [51]	3A4/5	[60]
Citalopram	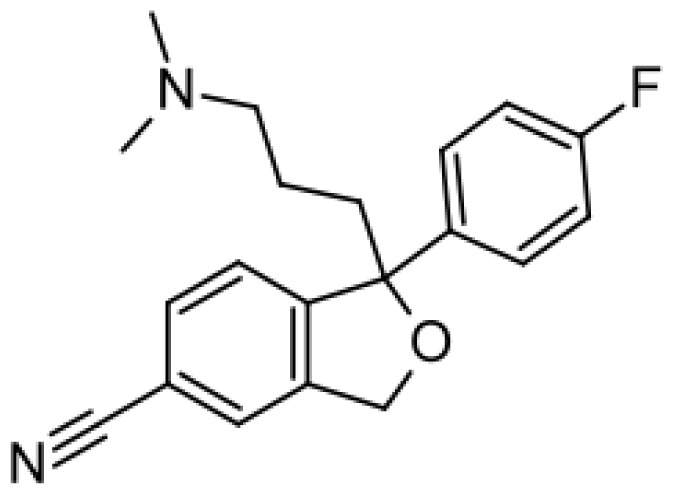	Demethylcitalopram [51]	3A4, 2C19	[61]
		di-demethylcitalopram [51]	2D6	
Clozapine	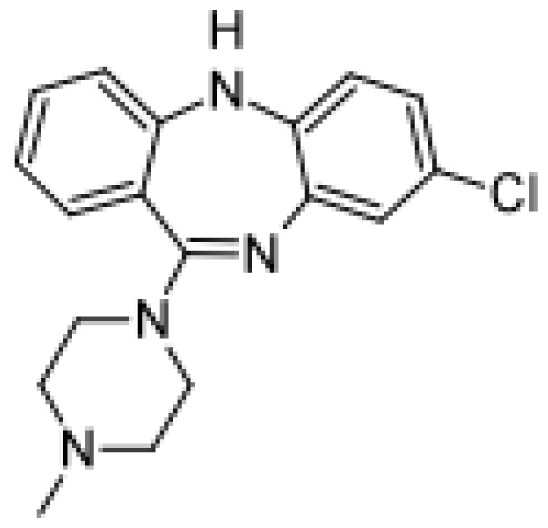	*N*-demthylclozapine [62]	1A2	[63]
		Clozapine *N*-oxide [62]	1A2	
Dextromethorphan	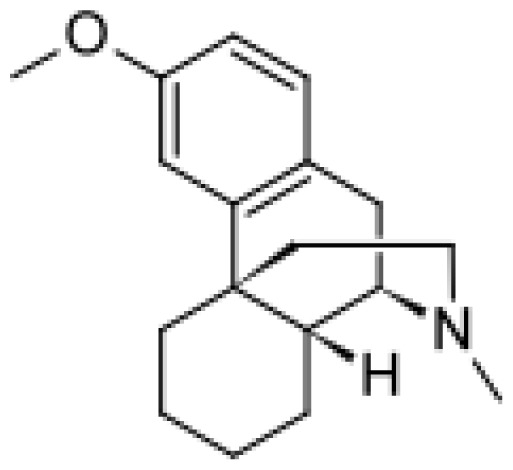	3-methoxymorphinan [49]	2D6, 3A4	[ 64]
Diclofenac	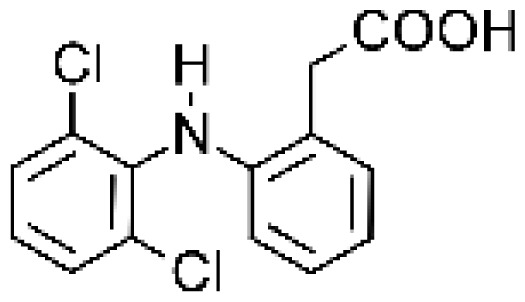	4′-hydroxydiclofenac [65, 66]	2C9/2C19	[67]
		5-hydroxydiclofenac [65]	2C9/2C19	
Diltiazem	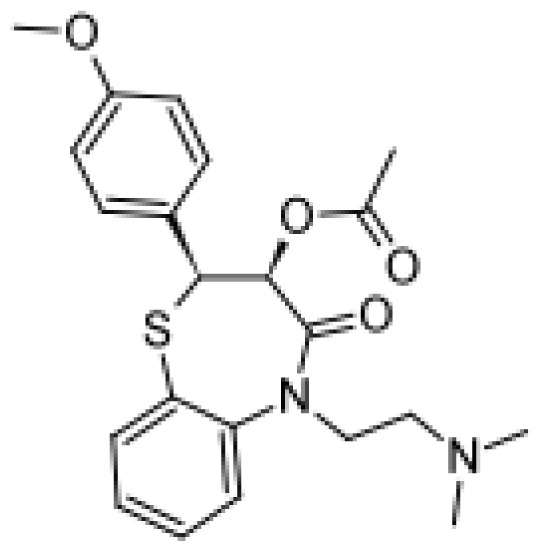	Demethyldiltiazem [51]	3A4	[68]
		Di-demethyldiltiazem [51]	3A4	
Irbesartan	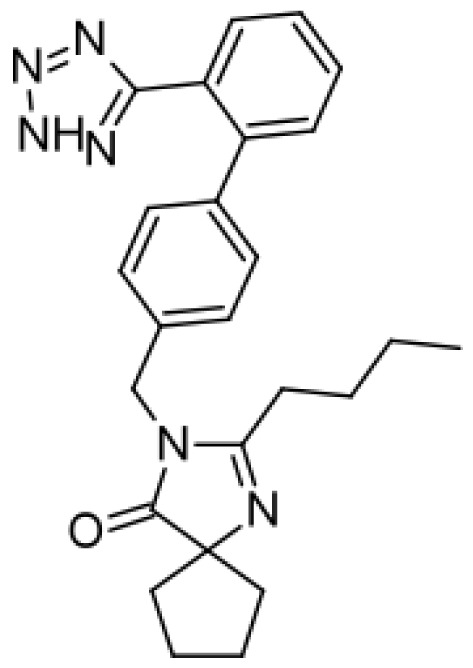	Hydroxyirbesartan [51]	2C9	[69]
		Di-hydroxyirbesartan [51]	2C9	
Lovastatin	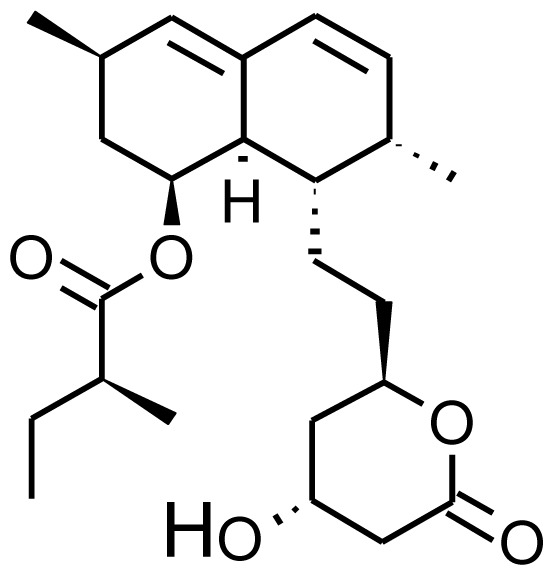	6b-hydroxylovastatin [70]	3A4/5	[71]
		6′-exomethylenelovastatin [70]	3A4/5	
Naproxen	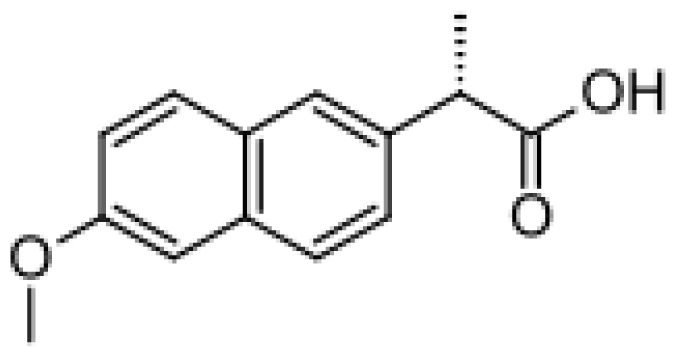	Desmethylnaproxen [47]	2C9, 1A2	[72]
Nifedipine	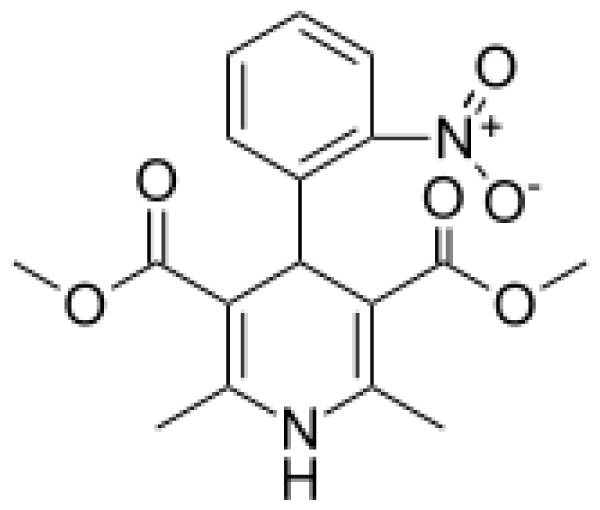	Oxidized nifedipine [37]	3A4	[73]
Ondansetron	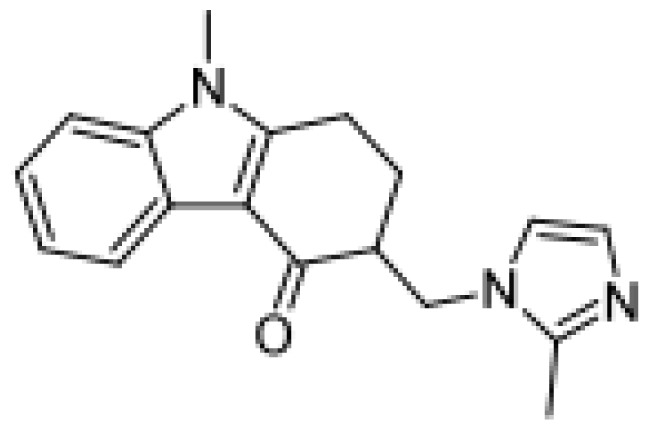	Hydroxylondansetron [51]	3A4, 2D6, 1A2	[74]
		Dihydroxyondansetron [51]	3A4, 2D6, 1A2	
Phenacetin	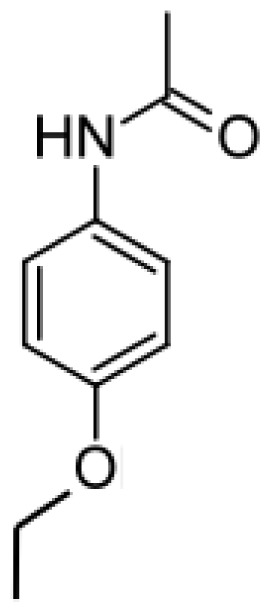	Acetaminophen [75]	1A2	[ 76]
Propafenone	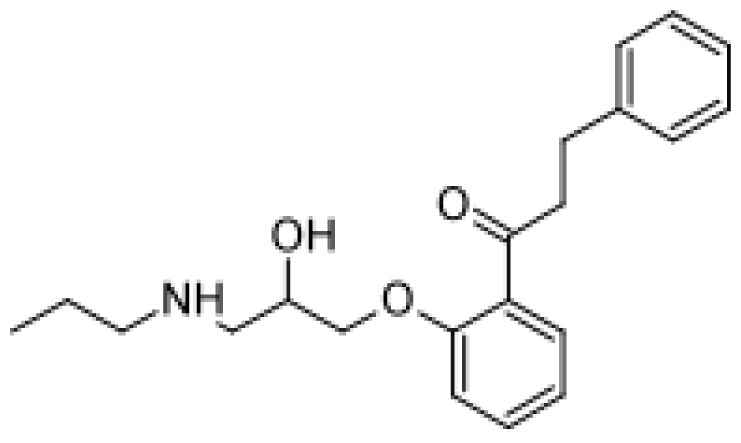	Hydroxylated metabolites [51]	2D6	[77]
		*N*-despropylpropafenone [51]	3A4/1A2	
Propranolol	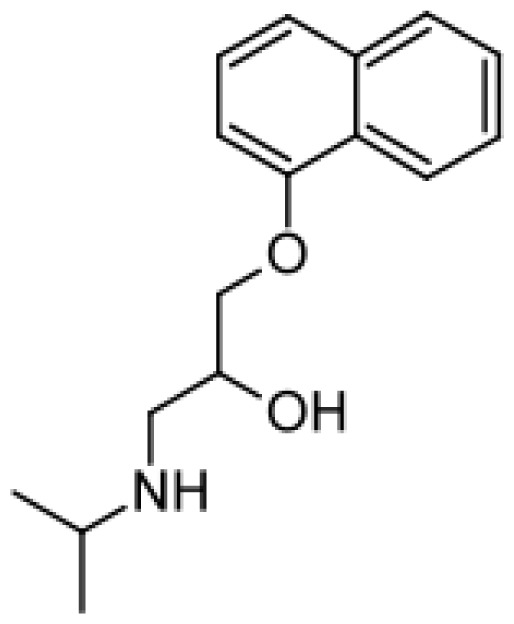	4′-hydroxypropranolol [78]	2D6	[79]
		5′-hydroxypropranolol [78]	2D6	
		*N*-despropylpropranolol [37, 78]	1A2	
Repaglinide	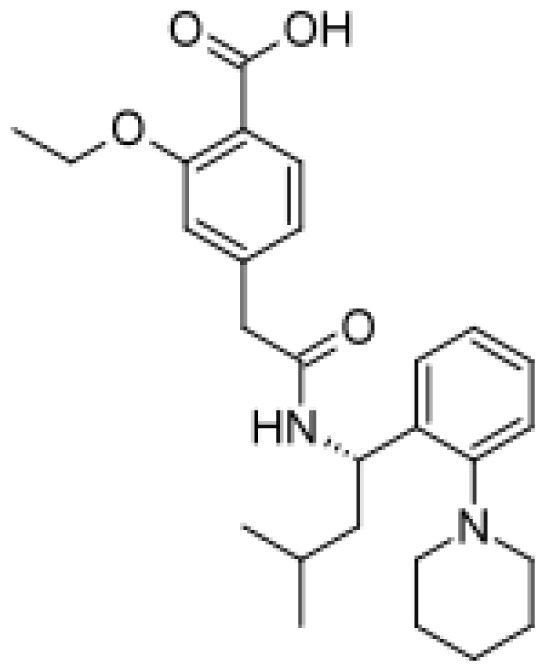	Hydroxylated metabolites [80]	3A4, 2C8	[81]
Simvastatin	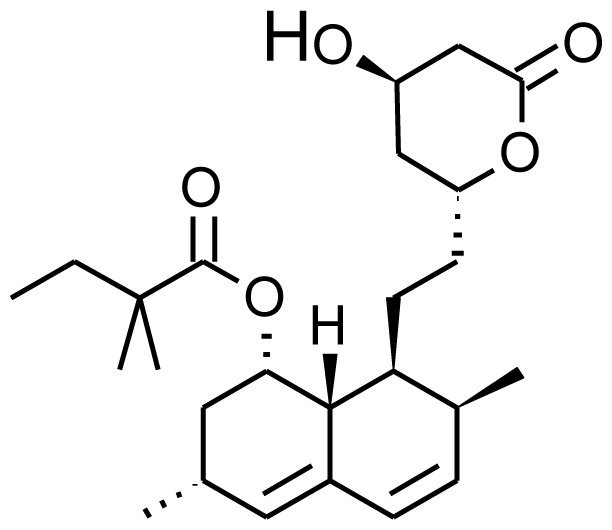	6b-hydroxysimvastatin [70]	3A4	[71]
		6′-exomethylenesimvastatin [70]	3A4	
Tolbutamide	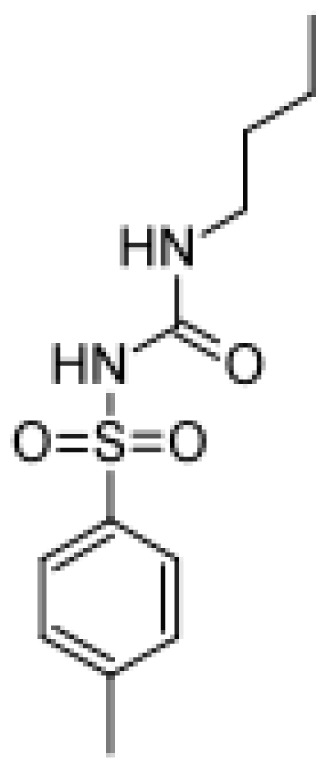	4-hydroxytolbutamide [37]	2C	[82]
Verapamil	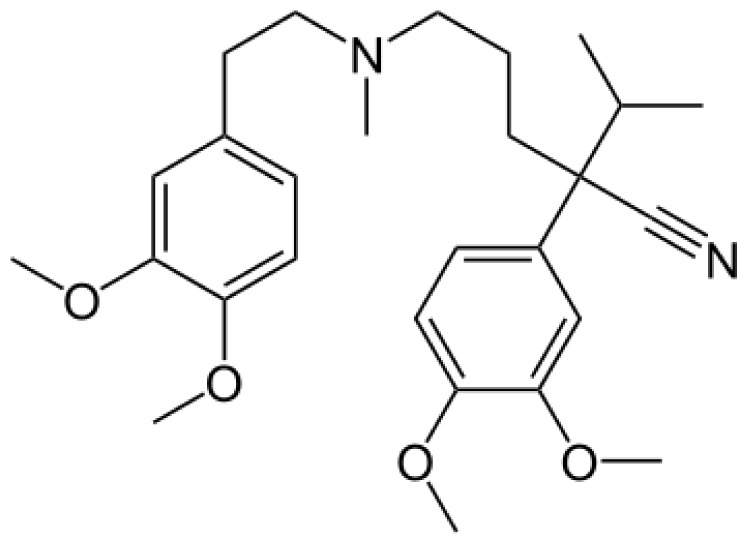	Norverapamil [ 55]	3A4	[83]
		D-617 [55]	3A4/3A5, 2C8	
		D-620 [55]	3A4/3A5, 2C8	
		D-702 [55]	2C9/2C18	
		PR-22 [55]	2C8	
		PR-25 [55]	2C8	

## References

[1] Kola I., Landis J. (2004). Can the pharmaceutical industry reduce attrition rates?. Nat. Rev. Drug Discovery.

[2] Singh S.S. (2006). Preclinical pharmacokinetics: An approach towards safer and efficacious drugs. Curr. Drug Metab.

[3] Fitzgerald J.D., O’Donnell S.R. (1971). Pharmacology of 4-hydroxypropranolol, a metabolite of propranolol. Br. J. Pharmacol.

[4] Coltart D.J., Shand D.G. (1970). Plasma propranolol levels in the quaniitative assessment of β-adrenergic blockade in man. Br. Med. J.

[5] Oatis J.E., Russell M.P., Knapp D.R., Walle T. (1981). Ring-hydroxylated propranolol: Synthesis and beta-receptor antagonist and vasodilating activities of the seven isomers. J. Med. Chem..

[6] Miller J.A. (1970). Carcinogenesis by chemicals: An overview—G. H. A. Clowes memorial lecture. Cancer Res.

[7] Miller J.A. (1994). Brief-history of chemical carcinogenesis. Cancer Lett.

[8] Guengerich F.P. (2005). Generation of reactive intermediates. J. Biochem. Mol. Toxicol.

[9] Mitchell J.R., Jollow D.J., Potter W.Z., Davis D.C., Gillette J.R., Brodie B.B. (1973). Acetaminophen-induced hepatic necrosis. I. Role of drug metabolism. J. Pharmacol. Exp. Ther.

[10] Dahlin D.C., Miwa G.T., Lu A.Y., Nelson S.D. (1984). *N*-acetyl-*p*-benzoquinone imine: A cytochrome P-450-mediated oxidation product of acetaminophen. Proc. Natl. Acad. Sci. USA.

[11] Markham A., Wagstaff A.J. (1998). Fexofenadine. Drugs.

[12] Ortiz de Montellano P.R., DeVoss J.J., Ortiz de Montellano P.R. (2005). CytochromeP450: Structure, Mechanism, and Biochemistry.

[13] Guengerich F.P. (2001). Common and uncommon cytochrome P450 reactions related to metabolism and chemical toxicity. Chem. Res. Toxicol.

[14] Lewis D.F., Sheridan G. (2001). Cytochromes P450, oxygen, and evolution. Sci. World J.

[15] Sono M., Roach M.P., Coulter E.D., Dawson J.H. (1996). Heme-containing oxygenases. Chem. Rev.

[16] Sakaguchi M., Mihara K., Sato R. (1987). A short amino-terminal segment of microsomal cytochrome-P-450 functions both as an insertion signal and as a stop transfer sequence. EMBO J.

[17] Black S.D. (1992). Membrane topology of the mammalian P450-cytochromes. FASEB J.

[18] Porter T.D., Kasper C.B. (1985). Coding nucleotide-sequence of rat nadph-cytochrome-P-450 oxidoreductase-cdna and identification of flavin-binding domains. Proc. Natl. Acad. Sci. USA.

[19] Bertz R.J., Granneman G.R. (1997). Use of *in vitro* and *in vivo* data to estimate the likelihood of metabolic pharmacokinetic interactions. Clin. Pharmacoket.

[20] Evans W.E., Relling M.V. (1999). Pharmacogenomics: Translating functional genomics into rational therapeutics. Science.

[21] Baranczewski P., Stanczak A., Kautiainen A., Sandin P., Edlund P.O. (2006). Introduction to early *in vitro* identification of metabolites of new chemical entities in drug discovery and development. Pharmacol. Rep.

[22] Fantuzzi A., Capria E., Mak L.H., Dodhia V.R., Sadeghi S.J., Collins S., Somers G., Huq E., Gilardi G. (2010). An electrochemical microfluidic platform for human p450 drug metabolism profiling. Anal. Chem.

[23] Parikh A., Gillam E.M.J., Guengerich F.P. (1997). Drug metabolism by *Escherichia coli* expressing human cytochromes P450. Nat. Biotechnol.

[24] Vail R.B., Homann M.J., Hanna I., Zaks A. (2005). Preparative synthesis of drug metabolites using human cytochrome P450s 3A4, 2C9 and 1A2 with NADPH-P450 reductase expressed in *Escherichia coli*. J. Ind. Microbiol. Biotechnol.

[25] Rushmore T.H., Reider P.J., Slaughter D., Assang C., Shou M. (2000). Bioreactor systems in drug metabolism: Synthesis of cytochrome P450-generated metabolites. Metab. Eng.

[26] Gillam E.M., Guengerich F.P. (2001). Exploiting the versatility of human cytochrome P450 enzymes: The promise of blue roses from biotechnology. IUBMB Life.

[27] Kumar S., Liu H., Halpert J.R. (2006). Engineering of cytochrome P450 3A4 for enhanced peroxide-mediated substrate oxidation using directed evolution and site-directed mutagenesis. Drug Metab. Dispos.

[28] Bernhardt R. (2006). Cytochromes P450 as versatile biocatalysts. J. Biotechnol.

[29] Lamb D.C., Waterman M.R., Kelly S.L., Guengerich F.P. (2007). Cytochromes P450 and drug discovery. Curr. Opin. Biotechnol.

[30] Gillam E.M. (2007). Extending the capabilities of nature’s most versatile catalysts: directed evolution of mammalian xenobiotic-metabolizing P450s. Arch. Biochem. Biophys.

[31] Kumar S. (2010). Engineering cytochrome P450 biocatalysts for biotechnology, medicine and bioremediation. Expert Opin. Drug Metab. Toxicol.

[32] Guengerich F.P. (2002). Cytochrome P450 enzymes in the generation of commercial products. Nat. Rev. Drug Discovery.

[33] Julsing M.K., Cornelissen S., Buehler B., Schmid A. (2008). Heme-iron oxygenases: Powerful industrial biocatalysts?. Curr. Opin. Chem. Biol.

[34] Nahri L.O., Fulco A.J. (1986). Characterization of a catalytically self-sufficient 119,000-dalton cytochrome P-450 monooxygenase induced by barbiturates in Bacillus megaterium. J. Biol. Chem.

[35] Noble M.A., Miles C.S., Chapman S.K., Lysek D.A., Mackay A.C., Reid G.A., Hanzlik R.P., Munro A.W. (1999). Roles of key active-site residues in flavocytochrome P450 BM3. Biochem. J.

[36] Munro A.W., Daff S., Coggins J.R., Lindsay J.G., Chapman S.K. (1996). Probing electron transfer in flavocytochrome P-450 BM3 and its component domains. Eur. J. Biochem.

[37] Di Nardo G., Fantuzzi A., Sideri A., Panicco P., Sassone C., Giunta C., Gilardi G. (2007). Wild-type CYP102A1 as a biocatalyst: Turnover of drugs usually metabolised by human liver enzymes. J. Biol. Inorg. Chem.

[38] Tsotsou G.E., Cass A.E.G., Gilardi G. (2002). High throughput assay for cytochrome P450BM3 for screening libraries of substrates and combinatorial mutants. Biosens. Bioelectron.

[39] Tsotsou G.E., di Nardo G., Sadeghi S.J., Fruttero R., Lazzarato L., Bertinaria M., Gilardi G. (2012). A rapid screening for cytochrome P450 catalysis on new chemical entities: Cytochrome P450 BM3 and 1,2,5-oxadiazole derivatives. J. Biomol. Screening.

[40] Yun C.H., Kim K.H., Kim D.H., Jung H.C., Pan J.G. (2007). The bacterial P450BM3: A prototype for a biocatalyst with human P450 activities. Trends Biotechnol.

[41] Kim D.H., Kim K.H., Kim D.H., Liu K.H., Jung H.C., Pan J.G., Yun C.H. (2008). Generation of Human Metabolites of 7-Ethoxycoumarin by Bacterial Cytochrome P450BM3. Drug Metab. Dispos.

[42] Kim D.H., Ahn T., Jung H.C., Pan J.G., Yun C.H. (2008). Generation of the human metabolite piceatannol from the anticancer-preventive agent resveratrol by bacterial cytochrome P450 BM3. Drug Metab. Dispos.

[43] Warman A.J., Roitel O., Neeli R., Girvan H.M., Seward H.E., Murray S.A., McLean K.J., Joyce M.G., Toogood H., Holt R.A. (2005). Flavocytochrome P450BM3: an update on structure and mechanism of a biotechnologically important enzyme. Biochem. Soc. Trans.

[44] McLean K.J., Girvan H.M., Munro A.W. (2007). Cytochrome P450/redox partner fusion enzymes: Biotechnological and toxicological prospects. Expert Opin. Drug Metab. Toxicol.

[45] Ravichandran K.G., Boddupalli S.S., Hasemann C.A., Peterson J.A., Deisenhofer J. (1993). Crystal-structure of hemoprotein domain of p450bm-3, a prototype for microsomal P450’s. Science.

[46] Li H.Y., Poulos T.L. (1997). The structure of the cytochrome P450BM-3 haem domain complexed with the fatty acid substrate, palmitoleic acid. Nat. Struct. Biol.

[47] Rentmeister A., Brown T.R., Snow C.D., Carbone M.N., Arnold F.H. (2011). Engineered bacterial mimics of human drug metabolizing enzyme CYP2C9. ChemCatChem.

[48] Van Vugt-Lussenburg B.M.A., Babel L.C., Vermeulen N.P.E., Commandeur J.N.M. (2005). Evaluation of alkoxyresorufins as fluorescent substrates for cytochrome P450BM3 and site-directed mutants. Anal. Biochem.

[49] Van Vugt-Lussenburg B.M.A., Damsten M.C., Maasdijk D.M., Vermeulen N.P.E., Commandeur J.N.M. (2006). Heterotropic and homotropic cooperativity by a drug-metabolising mutant of cytochrome P450BM3. Biochem. Biophys. Res. Commun.

[50] Patten C.J., Thomas P.E., Guy R.L., Lee M., Gonzalez F.J., Guengerich F.P., Yang C.S. (1993). Cytochrome-P450 enzymes involved in acetaminophen activation by rat and human liver-microsomes and their kinetics. Chem. Res. Toxicol.

[51] Reinen J., van Leeuwen J.S., Li Y., Sun L., Grootenhuis P.D., Decker C.J., Saunders J., Vermeulen N.P., Commandeur J.N. (2011). Efficient screening of cytochrome P450 BM3 mutants for their metabolic activity and diversity toward a wide set of drug-like molecules in chemical space. Drug Metab. Dispos.

[52] Wen B., Ma L., Zhu M. (2008). Bioactivation of the tricyclic antidepressant amitriptyline and its metabolite nortriptyline to arene oxide intermediates in human liver microsomes and recombinant P450s. Chemico-Biol. Interact.

[53] Van Vugt-Lussenburg B.M.A., Stjernschantz E., Lastdrager J., Oostenbrink C., Vermeulen N.P.E., Commandeur J.N.M. (2007). Identification of critical residues in novel drug metabolizing mutants of cytochrome P450BM3 using random mutagenesis. J. Med. Chem.

[54] Li X.Q., Bjorkman A., Andersson T.B., Ridderstrom M., Masimirembwa C.M. (2002). Amodiaquine clearance and its metabolism to *N*-desethylamodiaquine is mediated by CYP2C8: A new high affinity and turnover enzyme-specific probe substrate. J. Pharmacol. Exp. Ther.

[55] Sawayama A.M., Chen M.M.Y., Kulanthaivel P., Kuo M.S., Hemmerle H., Arnold F.H. (2009). A panel of cytochrome P450 BM3 variants to produce drug metabolites and diversify lead compounds. Chemistry.

[56] Matsumoto S., Yamazoe Y. (2001). Involvement of multiple human cytochromes P450 in the liver microsomal metabolism of astemizole and a comparison with terfenadine. Br. J. Clin. Pharmacol.

[57] Landwehr M., Hochrein L., Otey C.R., Kasrayan A., Backvall J.E., Arnold F.H. (2006). Enantioselective alpha-hydroxylation of 2-arylacetic acid derivatives and buspirone catalyzed by engineered cytochrome P450BM-3. J. Am. Chem. Soc.

[58] Zhu M.S., Zhao W.P., Jimenez H., Zhang D.L., Yeola S., Dai R.K., Vachharajani N., Mitroka J. (2005). Cytochrome P450 3A-mediated metabolism of buspirone in human liver microsomes. Drug Metab. Dispos.

[59] Peter R., Bocker R., Beaune P.H., Iwasaki M., Guengerich F.P., Yang C.S. (1990). Hydroxylation of chlorzoxazone as a specific probe for human liver cytochromeP-450IIE1. Chem. Res. Toxicol.

[60] Hiratsuka M., Hinai Y., Sasaki T., Konno Y., Imagawa K., Ishikawa M., Mizugaki M. (2007). Characterization of human cytochrome P450 enzymes involved in the metabolism of cilostazol. Drug Metab. Dispos.

[61] Brosen K., Naranjo C.A. (2001). Review of pharmacokinetic and pharmacodynamic interaction studies with citalopram. Eur. Neuropsychopharmacol.

[62] Damsten M.C., van Vugt-Lussenburg B.M.A., Zeldenthuis T., de Vlieger J.S.B., Commandeur J.N.M., Vermeulen N.P.E. (2008). Application of drug metabolising mutants of cytochrome P450BM3 (CYP102A1) as biocatalysts for the generation of reactive metabolites. Chemico-Biol. Interact.

[63] Urichuk L., Prior T.I., Dursun S., Baker G. (2008). Metabolism of atypical antipsychotics: involvement of cytochrome P450 enzymes and relevance for drug-drug interactions. Curr. Drug Metab.

[64] Yu A.M., Haining R.L. (2001). Comparative contribution to dextromethorphan metabolism by cytochrome P450 isoforms *in vitro*: Can dextromethorphan be used as a dual probe for both CYP2D6 and CYP3A activities?. Drug Metab. Dispos.

[65] Van Leeuwen J.S., Vredenburg G., Dragovic S., Tjong T.F.J., Vos J.C., Vermeulen N.P.E. (2011). Metabolism related toxicity of diclofenac in yeast as model system. Toxicol. Lett.

[66] Tsotsou G.E., Sideri A., Goyal A., di Nardo G., Gilardi G. (2012). Identification of mutant Asp251Gly/Gln307His of cytochrome P450 BM3 for the generation of metabolites of diclofenac, ibuprofen and tolbutamide. Chemistry.

[67] Mancy A., Antignac M., Minoletti C., Dijols S., Mouries V., Duong N.T.H., Battioni P., Dansette P.M., Mansuy D. (1999). Diclofenac and its derivatives as tools for studying human cytochromes P450 active sites: Particular efficiency and regioselectivity of P4502Cs. Biochemistry.

[68] Pinto A.G., Horlander J., Chalasani N., Hamman M., Asghar A., Kolwankar D., Hall S.D. (2005). Diltiazem inhibits human intestinal cytochrome P450 3A (CYP3A) activity *in vivo* without altering the expression of intestinal mRNA or protein. Br. J. Clin. Pharmacol.

[69] Bourrie M., Meunier V., Berger Y., Fabre G. (1999). Role of cytochrome P-4502C9 in irbesartan oxidation by human liver microsomes. Drug Metab. Dispos.

[70] Kim K.H., Kang J.Y., Kim D.H., Park S.H., Park S.H., Kim D., Park K.D., Lee Y.J., Jung H.C., Pan J.G. (2011). Generation of human chiral metabolites of simvastatin and lovastatin by bacterial CYP102A1 mutants. Drug Metab. Dispos.

[71] Garcia M.J., Reinoso R.F., Navarro A.S., Prous J.R. (2003). Clinical pharmacokinetics of statins. Methods Findings Exp. Clin. Pharmacol.

[72] Miners J.O., Coulter S., Tukey R.H., Veronese M.E., Birkett D.J. (1996). Cytochromes P450, 1A2, and 2C9 are responsible for the human hepatic *O*-demethylation of *R*- and *S*-naproxen. Biochem. Pharmacol.

[73] Guengerich F.P., Martin M.V., Beaune P.H., Kremers P., Wolff T., Waxman D.J. (1986). Characterization of rat and human-liver microsomal cytochrome-P-450 forms involved in nifedipine oxidation, a prototype for genetic-polymorphism in oxidative drug-metabolism. J. Biol. Chem.

[74] Dixon C.M., Colthup P.V., Serabjit-Singh C.J., Kerr B.M., Boehlert C.C., Park G.R., Tarbit M.H. (1995). Multiple forms of cytochrome-P450 are involved in the metabolism of ondansetron in humans. Drug Metab. Dispos.

[75] Park S.H., Kim D.H., Kim D., Jung H.C., Pan J.G., Ahn T., Yun C.H. (2010). Engineering bacterial cytochrome P450 (P450) BM3 into a prototype with human P450 enzyme activity using indigo formation. Drug Metab. Dispos.

[76] Yun C.H., Miller G.P., Guengerich F.P. (2000). Rate-determining steps in phenacetin oxidations by human cytochrome P450 1A2 and selected mutants. Biochemistry.

[77] Botsch S., Gautier J.C., Beaune P., Eichelbaum M., Kroemer H.K. (1993). Identification and characterization of the cytochrome-P450 enzymes involved in *n*-dealkylation of propafenone-molecular-base for interaction potential and variable disposition of active metabolites. Mol. Pharmacol.

[78] Otey C.R., Bandara G., Lalonde J., Takahashi K., Arnold F.H. (2006). Preparation of human metabolites of propranolol using laboratory-evolved bacterial cytochromes P450. Biotechnol. Bioeng.

[79] McGinnity D.F., Parker A.J., Soars M., Riley R.J. (2000). Automated definition of the enzymology of drug oxidation by the major human drug metabolizing cytochrome P450s. Drug Metab. Dispos.

[80] Meuldermans W., Hendrickx J., Lauwers W., Hurkmans R., Swysen E., Heykants J. (1986). Excretion and biotransformation of astemizole in rats, guinea-pigs, dogs, and man. Drug Dev. Res.

[81] Bidstrup T.B., Bjornsdottir I., Sidelmann U.G., Thomsen M.S., Hansen K.T. (2003). CYP2C8 and CYP3A4 are the principal enzymes involved in the human *in vitro* biotransformation of the insulin secretagogue repaglinide. Br. J. Clin. Pharmacol.

[82] Transon C., Leemann T., Dayer P. (1996). *In vitro* comparative inhibition profiles of major human drug metabolising cytochrome P450 isozymes (CYP2C9, CYP2D6 and CYP3A4) by HMG-CoA reductase inhibitors. Eur. J. Clin. Pharmacol.

[83] Tracy T.S., Korzekwa K.R., Gonzalez F.J., Wainer I.W. (1999). Cytochrome P450 isoforms involved in metabolism of the enantiomers of verapamil and norverapamil. Br. J. Clin. Pharmacol.

[84] Rea V., Kolkman A.J., Vottero E., Stronks E.J., Ampt K.A.M., Honing M., Vermeulen N.P.E., Wijmenga S.S., Commandeur J.N.M. (2012). Active site substitution A82W improves the regioselectivity of steroid hydroxylation by cytochrome P450 BM3 mutants as rationalized by spin relaxation nuclear magnetic resonance studies. Biochemistry.

[85] Venkataraman H., de Beer S.B.A., van Bergen L.A.H., van Essen N., Geerke D.P., Vermeulen N.P.E., Commandeur J.N.M. (2012). A single active site mutation inverts stereoselectivity of 16-hydroxylation of testosterone catalyzed by engineered cytochrome P450 BM3. ChemBioChem.

[86] Laine L., Goldkind L., Curtis S.P., Connors L.G., Zhang Y., Cannon C.P. (2009). How common is diclofenac-associated liver injury? Analysis of 17,289 arthritis patients in a long-term prospective clinical trial. Am. J. Gastroenterol.

[87] McTavish D., Sorkin E.M. (1989). Verapamil—An updated review of its pharmacodynamic and pharmacokinetic properties, and therapeutic use in hypertension. Drugs.

[88] Peters M.W., Meinhold P., Glieder A., Arnold F.H. (2003). Regio- and enantioselective alkane hydroxylation with engineered cytochromes P450 BM-3. J. Am. Chem. Soc.

[89] Meinhold P., Peters M.W., Hartwick A., Hernandez A.R., Arnold F.H. (2006). Engineering cytochrome P450BM3 for terminal alkane hydroxylation. Adv. Synth. Catal.

[90] Kroemer H.K., Gautier J.C., Beaune P., Henderson C., Wolf C.R., Eichelbaum M. (1993). Identification of P450 enzymes involved in metabolism of verapamil in humans. Naunyn-Schmiedebergs Arch. Pharm.

[91] Vlahos C.J., Matter W.F., Hui K.Y., Brown R.F. (1994). A specific inhibitor of phosphatidylnositol 3-kinase, 2-(4-morpholinyl)-8-phenyl-4*H*-1-benzopyran-4-one (LY294002). J. Biol. Chem.

[92] Van der Weide J., Steijns L.S.W., van Weelden M.J.M. (2003). The effect of smoking and cytochrome P450CYP1A2 genetic polymorphism on clozapine clearance and dose requirement. Pharmacogenetics.

[93] Cowart L.A., Falck J.R., Capdevila J.H. (2001). Structural determinants of active site binding affinity and metabolism by cytochrome P450BM-3. Arch. Biochem. Biophys.

[94] Haines D.C., Hegde A., Chen B., Zhao W., Bondlela M., Humphreys J.M., Mullin D.A., Tomchick D.R., Machius M., Peterson J.A. (2011). A single active-site mutation of P450BM-3 dramatically enhances substrate binding and rate of product formation. Biochemistry.

[95] Hunter D.J.B., Behrendorff J.B.Y.H., Johnston W.A., Hayes P.Y., Huang W., Bonn B., Hayes M.A., de Voss J.J., Gillam E.M.J. (2011). Facile production of minor metabolites for drug development using a CYP3A shuffled library. Metab. Eng.

[96] Rosic N.N., Huang W., Johnston W.A., DeVoss J.J., Gillam E.M.J. (2007). Extending the diversity of cytochrome P450 enzymes by DNA family shuffling. Gene.

[97] Johnston W.A., Huang W., de Voss J.J., Hayes M.A., Gillam E.M.J. (2007). A shuffled CYP1A library shows both structural integrity and functional diversity. Drug Metab. Dispos.

[98] Huang W., Johnston W.A., Hayes M.A., de Voss J.J., Gillam E.M.J. (2007). A shuffled CYP2C library with a high degree of structural integrity and functional versatility. Arch. Biochem. Biophys.

[99] Rosic N.N. (2009). Versatile capacity of shuffled cytochrome P450s for dye production. Appl. Microbiol. Biotechnol.

[100] Weis R., Winkler M., Schittmayer M., Kambourakis S., Vink M., Rozzell J.D., Glieder A. (2009). A diversified library of bacterial and fungal bifunctional cytochrome P450 enzymes for drug metabolite synthesis. Adv. Synth. Catal.

[101] Otey C.R., Silberg J.J., Voigt C.A., Endelman J.B., Bandara G., Arnold F.H. (2004). Functional evolution and structural conservation in chimeric cytochromes P450: Calibrating a structure-guided approach. Chem. Biol.

[102] Gilardi G., Meharenna Y.T., Tsotsou G.E., Sadeghi S.J., Fairhead M., Giannini S. (2002). Molecular Lego: Design of molecular assemblies of P450 enzymes for nanobiotechnology. Biosens. Bioelectron.

[103] Bernhardt R. (2004). Optimized chimeragenesis: Creating diverse P450 functions. Chem. Biol.

[104] Sadeghi S.J., Ferrero S., di Nardo G., Gilardi G. (2012). Drug-drug interactions and cooperative effects detected in electrochemically driven human cytochrome P450 3A4. Bioelectrochemistry.

[105] Denisov I.G., Sligar S.G. (2012). A novel type of allosteric regulation: Functional cooperativity in monomeric proteins. Arch. Biochem. Biophys.

[106] Roberts A.G., Yang J., Halpert J.R., Nelson S.D., Thummel K.T., Atkins W.M. (2011). The structural basis for homotropic and heterotropic cooperativity of midazolam metabolism by human cytochrome P450 3A4. Biochemistry.

[107] Woods C.M., Fernandez C., Kunze K.L., Atkins W.M. (2011). Allosteric activation of cytochrome P450 3A4 by alpha-naphthoflavone: Branch point regulation revealed by isotope dilution analysis. Biochemistry.

[108] Fernando H., Rumfeldt J.A.O., Davydova N.Y., Halpert J.R., Davydov D.R. (2011). Multiple substrate-binding sites are retained in cytochrome P450 3A4 mutants with decreased cooperativity. Xenobiotica.

[109] Frank D.J., Denisov I.G., Sligar S.G. (2009). Mixing apples and oranges: Analysis of heterotropic cooperativity in cytochrome P450 3A4. Arch. Biochem. Biophys.

[110] Frank D.J., Denisov I.G., Sligar S.G. (2011). Analysis of heterotropic cooperativity in cytochrome P450 3A4 using alpha-naphthoflavone and testosterone. J. Biol. Chem.

[111] Perret A., Pompon D. (1998). Electron shuttle between membrane-bound cytochrome P450 3A4 and b(5) rules uncoupling mechanisms. Biochemistry.

[112] Whitehouse C.J.C., Bell S.G., Wong L.-L. (2012). P450(BM3) (CYP102A1): Connecting the dots. Chem. Soc. Rev.

[113] Joo H., Lin Z.L., Arnold F.H. (1999). Laboratory evolution of peroxide-mediated cytochrome P450 hydroxylation. Nature.

[114] Cirino P.C., Arnold F.H. (2002). Regioselectivity and activity of cytochrome P450 BM-3 and mutant F87A in reactions driven by hydrogen peroxide. Adv. Synth. Catal.

[115] Cirino P.C., Arnold F.H. (2003). A self-sufficient peroxide-driven hydroxylation biocatalyst. Angew. Chem. Int. Ed. Engl.

[116] Ryan J.D., Fish R.H., Clark D.S. (2008). Engineering cytochrome p450 enzymes for improved activity towards biomimetic 1,4-NADH cofactors. ChemBioChem.

[117] Fleming B.D., Tian Y., Bell S.G., Wong L.L., Urlacher V., Hill H.A.O. (2003). Redox properties of cytochrome P450(BM3) measured by direct methods. Eur. J. Biochem.

[118] Udit A.K., Hill M.G., Bittner V.G., Arnold F.H., Gray H.B. (2004). Reduction of dioxygen catalyzed by pyrene-wired heme domain cytochrome P450BM3 electrodes. J. Am. Chem. Soc.

[119] Udit A.K., Hindoyan N., Hill M.G., Arnold F.H., Gray H.B. (2005). Protein-surfactant film voltammetry of wild-type and mutant cytochrome P450BM3. Inorg. Chem.

[120] Fantuzzi A., Meharenna Y.T., Briscoe P.B., Sassone C., Borgia B., Gilardi G. (2006). Improving catalytic properties of P450BM3 haem domain electrodes by molecular Lego. Chem. Commun.

[121] Fantuzzi A., Fairhead M., Gilardi G. (2004). Direct electrochemistry of immobilized human cytochrome P450 2E1. J. Am. Chem. Soc.

[122] Sadeghi S.J., Fantuzzi A., Gilardi G. (2011). Breakthrough in P450 bioelectrochemistry and future perspectives. Biochim. Biophys. Acta.

[123] Dodhia V.R., Sassone C., Fantuzzi A., di Nardo G., Sadeghi S.J., Gilardi G. (2008). Modulating the coupling efficiency of human cytochrome P450 CYP3A4 at electrode surfaces through protein engineering. Electrochem. Commun.

[124] Holtmann D., Mangold K.-M., Schrader J. (2009). Entrapment of cytochrome P450 BM-3 in polypyrrole for electrochemically-driven biocatalysis. Biotechnol. Lett.

[125] Schwaneberg U., Appel D., Schmitt J., Schmid R.D. (2000). P450 in biotechnology: zinc driven omega-hydroxylation of *p*-nitrophenoxydodecanoic acid using P450BM-3 F87A as a catalyst. J. Biotechnol.

[126] Hollmann F., Hofstetter K., Schmid A. (2006). Non-enzymatic regeneration of nicotinamide and flavin cofactors for monooxygenase catalysis. Trends Biotechnol.

[127] Salazar O., Cirino P.C., Arnold F.H. (2003). Thermostabilization of a cytochrome P450 peroxygenase. ChemBioChem.

[128] Li Y., Drummond D.A., Sawayama A.M., Snow C.D., Bloom J.D., Arnold F.H. (2007). A diverse family of thermostable cytochrome P450s created by recombination of stabilizing fragments. Nat. Biotechnol.

[129] Maurer S.C., Schulze H., Schmid R.D., Urlacher V. (2003). Immobilisation of P450BM-3 and an NADP(+) cofactor recycling system: Towards a technical application of heme-containing monooxygenases in fine chemical synthesis. Adv. Synth. Catal.

[130] Weber E., Sirim D., Schreiber T., Thomas B., Pleiss J., Hunger M., Glaeser R., Urlacher V.B. (2010). Immobilization of P450 BM-3 monooxygenase on mesoporous molecular sieves with different pore diameters. J. Mol. Catal. B.

[131] Zhao L., Gueven G., Li Y., Schwaneberg U. (2011). First steps towards a Zn/Co(III)sep-driven P450 BM3 reactor. Appl. Microbiol. Biotechnol.

[132] Yim S.K., Kim D.H., Jung H.C., Pan J.G., Kang H.S., Ahn T., Yun C.H. (2010). Surface display of heme- and diflavin-containing cytochrome P450 BM3 in *Escherichia coli*: A whole-cell biocatalyst for oxidation. J. Microbiol. Biotechnol.

[133] Schewe H., Holtmann D., Schrader J. (2009). P450(BM-3)-catalyzed whole-cell biotransformation of alpha-pinene with recombinant *Escherichia coli* in an aqueous-organic two-phase system. Appl. Microbiol. Biotechnol.

[134] Lu Y., Mei L.H. (2007). Optimization of fermentation conditions for P450 BM-3 monooxygenase production by hybrid design methodology. J. Zhejiang Univ. Sci.

[135] Pflug S., Richter S.M., Urlacher V.B. (2007). Development of a fed-batch process for the production of the cytochrome P450 monooxygenase CYP102A1 from *Bacillus megaterium* in *E. coli*. J. Biotechnol.

[136] Park J.W., Lee J.K., Kwon T.J., Yi D.H., Kim Y.J., Moon S.H., Suh H.H., Kang S.M., Park Y.I. (2003). Bioconversion of compactin into pravastatin by *Streptomyces* sp. Biotechnol. Lett.

